# Responses of CH_4_ and N_2_O fluxes to land-use conversion and fertilization in a typical red soil region of southern China

**DOI:** 10.1038/s41598-017-10806-z

**Published:** 2017-09-05

**Authors:** Xing Wu, Huifeng Liu, Xunhua Zheng, Fei Lu, Shuai Wang, Zongshan Li, Guohua Liu, Bojie Fu

**Affiliations:** 1 State Key Laboratory of Urban and Regional Ecology, Research Center for Eco-Environmental Sciences, Chinese Academy of Sciences, Beijing, 100085 China; 2 Joint Center for Global Change Studies, Beijing, 100875 China; 3 University of Chinese Academy of Science, Beijing, 100049 China; 4 State Key Laboratory of Atmospheric Boundary Layer Physics and Atmospheric Chemistry, Institute of Atmospheric Physics, Chinese Academy of Sciences, Beijing, 100029 China

## Abstract

Land-use conversion and fertilization have been widely reported as important management practices affecting CH_4_ and N_2_O fluxes; however, few long-term *in situ* measurements are available after land-use conversion from rice paddies to upland cultivation, especially those including the initial stages after conversion. A 3-year field experiment was conducted in rice paddies and a newly converted citrus orchard to measure CH_4_ and N_2_O fluxes in response to land-use conversion and fertilization in a red soil region of southern China. Annual CH_4_ and N_2_O emissions averaged 303.9 kg C ha^−1^ and 3.8 kg N ha^−1^, respectively, for the rice paddies over three cultivation years. Although annual N_2_O emissions increased two- to threefold after the conversion of rice paddies to citrus orchard, the substantial reduction in CH_4_ emissions and even shift into a sink for atmospheric CH_4_ led to significantly lower CO_2_-eq emissions of CH_4_ and N_2_O in the citrus orchard compared to the rice paddies. Moreover, distinct CH_4_ emissions were observed during the initial stages and sustained for several weeks after conversion. Our results indicated that the conversion of rice paddies to citrus orchards in this region for higher economic benefits may also lead to lower aggregate CH_4_ and N_2_O emissions.

## Introduction

The anthropogenic trace gases methane (CH_4_) and nitrous oxide (N_2_O), two major potent and long-lived greenhouse gases (GHGs), have 34 and 298 times higher radiative forcing, respectively, than CO_2_ over a time horizon of 100 years^1^. Agriculture ecosystem is one of the major sources for these anthropogenic emissions, accounting for approximately 50% and 60% of the total global CH_4_ and N_2_O emissions, respectively^2^. Paddy fields, in particular, have been well documented as a significant source of atmospheric CH_4_ and can release substantial N_2_O. The periodic waterlogging-drainage alteration episodes and intensive inputs of organic material and nitrogen fertilizer in paddy fields may provide a suitable soil environment and accessible substrate for CH_4_ and N_2_O emissions^3, 4^. Many studies have demonstrated high CH_4_ but relatively low N_2_O emissions from rice paddies because anaerobic conditions limit nitrate availability and because strict anaerobiosis favours complete denitrification to nitrogen gas (N_2_)^5, 6^. However, N_2_O emissions are generally high in upland soils, especially after fertilization or irrigation events, due to the tight coupling between nitrification and denitrification^7–9^. Therefore, the conversion of rice paddies to upland agriculture might result in ‘pollution swapping’, that is, reduced CH_4_ emissions at the expense of an increase in N_2_O emissions, due to changes in soil environmental conditions and management practices^9, 10^.

Land-use change, which is regarded as the second largest anthropogenic source of greenhouse gas emissions, can substantially alter the dynamics of soil gases^11–13^. However, land-use change can also decrease, increase, or have no significant impact on soil CH_4_ and N_2_O fluxes^9, 13–16^. The high variability of soil CH_4_ and N_2_O fluxes due to land-use change is associated with particular site conditions, such as the soil type and microclimate, the type and history of land-use change, and the management practices used^16–18^. In general, conversion from rice paddy to upland agriculture can significantly reduce CH_4_ emissions or even convert the soil from an emission source to a sink for atmospheric CH_4_^6, 9^. However, when and to what extent can the soil act as an atmospheric CH_4_ sink after these land-use conversions still remain unclear. Moreover, most of the existing studies have focused on comparisons of different types of land uses that have been converted for many years^18–21^. Thus, little information is available for understanding the dynamics of CH_4_ and N_2_O fluxes and the underlying mechanisms involved during the initial stages after land-use conversion.

China is one of the most important rice-producing countries in the world, accounting for 20% of the global rice production area^6, 22^. The annual totals for CH_4_ and N_2_O emissions from Chinese rice paddies were approximately 4.5–7.5 Tg C yr^−1^ and 32–51 Gg N yr^−1^, respectively, based on long-term field measurements and model simulations^22–25^. Red soil, one of the typical agricultural soils in subtropical China, covers approximately 11.8% of the country’s land surface, producing 80% of the rice, and supporting 22.5% of the population of China^26^. During the past decades, the red soil regions, which are the most densely populated, have experienced significant changes in land use due to increased socio-economic development and demand for livestock products. In particular, conversion of rice paddies to upland cultivation for growing vegetables, fruits and economic forest has been locally advocated to meet increasing market demands and gain higher economic returns in these regions^6, 18^. Such conversions not only can alter the physical, chemical and biological properties of the soil, but also can impact on the soil C and N turnover and GHG emissions. However, detailed long-term measurements of combined CH_4_ and N_2_O fluxes during such conversions are still limited, and the impact of environmental factors and management practices on CH_4_ and N_2_O fluxes during such conversions are not fully understood, especially for the red soil regions in China.

Therefore, over a 3-year period, we conducted *in situ* measurements of CH_4_ and N_2_O fluxes from conventional paddy fields and a citrus plantation recently converted from paddy field in a typical hilly, red soil region of southern China. The main objectives of this study were to investigate the response characteristics and temporal changes of CH_4_ and N_2_O fluxes during land-use conversion from paddy fields to a citrus plantation and to assess the impact of environmental factors and fertilization on CH_4_ and N_2_O fluxes. Eventually, this study also attempted to examine whether the conversion of rice paddies to citrus plantation shows potential for mitigating CH_4_ and N_2_O emissions.

## Results

### Weather conditions and soil properties

**Figure 1:**
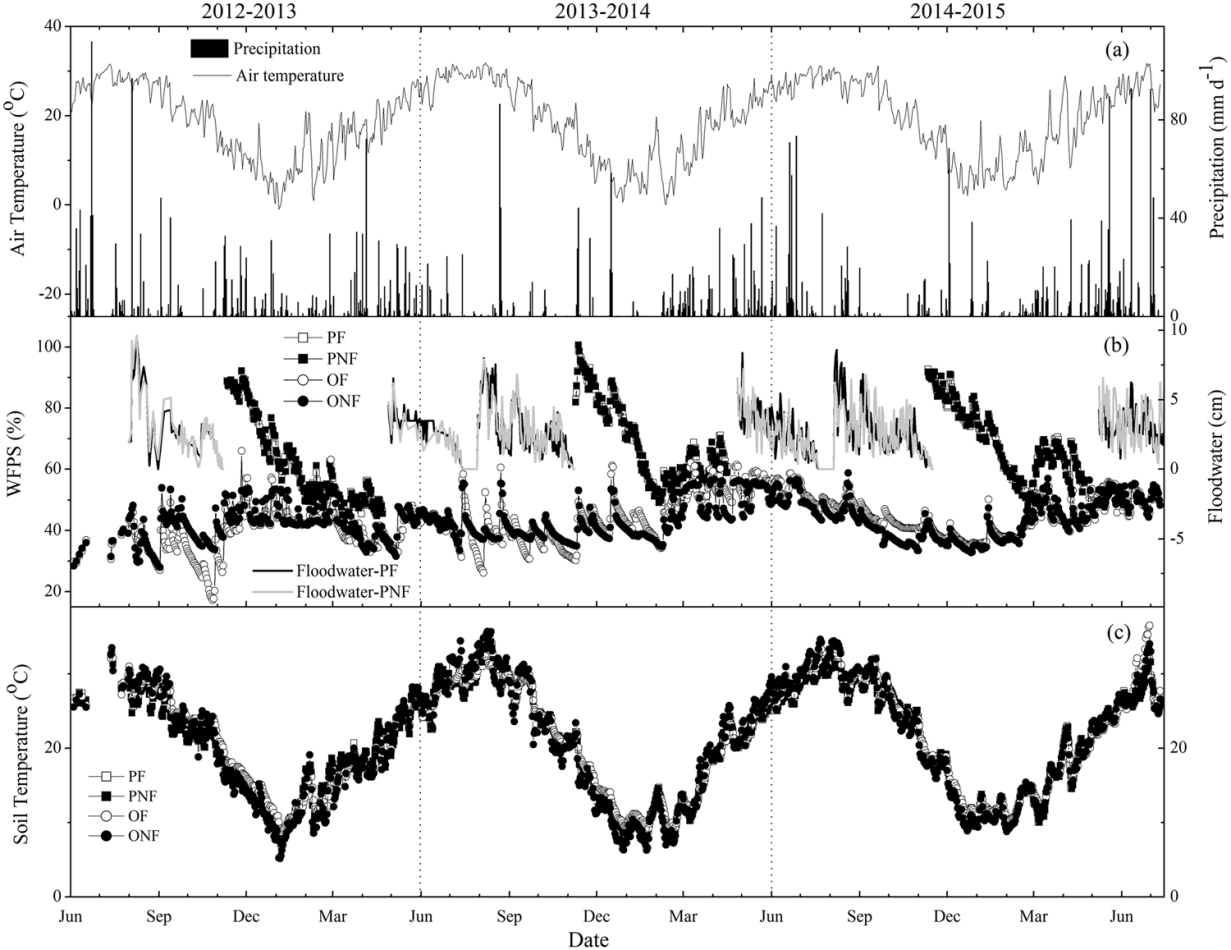
The dynamics of daily precipitation and air temperature (**a**), floodwater depth in the rice growing seasons and WFPS (water-filled pore space) at 0–10 cm soil depth in the non-rice seasons (**b)** and soil temperatures at 0–10 cm depth (**c**) from June 2012 to July 2015.

The annual mean air temperatures were 18.5, 18.6 and 18.9 °C for the 2012/2013, 2013/2014 and 2014/2015 cultivation years, respectively. The annual precipitation was lower in 2013/2014 (1117.6 mm) and 2014/2015 (1421.0 mm) cultivation years and higher in 2012/2013(1611.8 mm) compared to the long-term site average (1509.0 mm), and more than 70% of the rainfall occurred between March and August (Fig. 1a). Soil temperature showed a temporal pattern similar to that of air temperature for the three consecutive cultivation years (Fig. 1c). Land-use conversion from rice paddy field to a citrus orchard slightly increased the soil temperature but significantly reduced the soil moisture (Fig. 1b). However, fertilization did not significantly affect the soil temperature and moisture for either land-use type during the entire measurement period.

**Figure 2:**
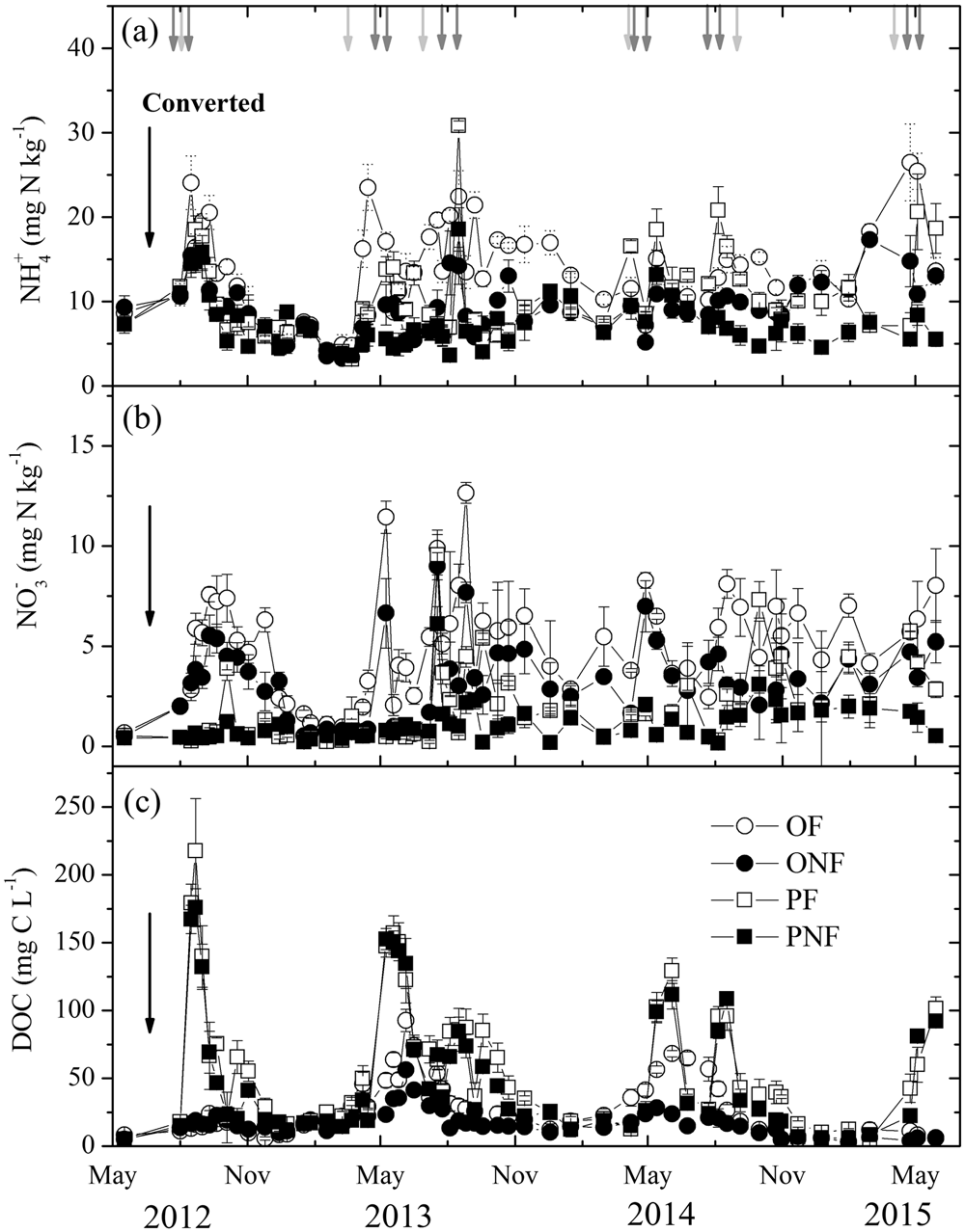
The dynamics of soil NH_4_^+^−N (**a**), NO_3_^–^−N (**b**) and DOC (**c**) concentrations in the four treatments from 2012 to 2015. The data are shown as the means with standard errors. The black arrow indicates the cultivation of orchard and paddy. The grey arrows indicate fertilizer applied to PF, and the light grey arrows indicate fertilizer applied to OF. The arrows in panel (**a**) applied to panel (**b**) and (**c**) as well.

As expected, land-use conversion and fertilization significantly changed the soil properties (Table 1 and Fig. 2). Before land-use conversion (May 2012), all the measured soil properties were not significantly different among different treatments (Table 1). However, the soil bulk density, TN and inorganic N (NH_4_^+^ and NO_3_^−^) content substantially increased three years after land-use conversion from rice paddy fields to a citrus orchard (May 2015), whereas the soil pH and DOC values significantly decreased (*P* < 0.05). Seasonal variations in the soil inorganic N content over the entire observation period were basically regulated by N application (Fig. 2a,b). The relatively high NH_4_^+^ and NO_3_^−^ contents were primarily observed within 10 days after fertilization. Overall, fertilization significantly increased (*P* < 0.05) the NH_4_^+^ and NO_3_^−^ contents for both land-use types. The dynamics of soil DOC concentration were mainly affected by irrigation and fertilization activities, especially for the rice paddy (Fig. 2c). Soil DOC concentrations in the fertilized rice paddy plots were significantly higher compared to the control (*P* < 0.05); however, the highest soil DOC values in the rice paddy gradually decreased with successive cultivation years (Fig. 2c).

**Table 1:** Main soil properties (0–10 cm) in study sites before and after land conversion. Data are shown as the means with standard errors for four spatial replicates. Different letters in the same column indicate significant differences (*P* < 0.05) between corresponding treatments. OF = orchard with fertilization, ONF = orchard without fertilization, PF = paddy with fertilization, and PNF = paddy without fertilization.

Treatments	pH	Bulk density (g cm^−3^)	Total nitrogen (TN, g kg^−1^)	Dissolved organic carbon (DOC, mg L^−1^)	NH_4_^+^−N (mg kg^−1^)	NO_3_^–^−N (mg kg^−1^)
**Before land-use conversion (May 2012)**						
OF	5.04 ± 0.05 a	1.28 ± 0.04 a	1.03 ± 0.02 a	6.06 ± 1.25 a	8.63 ± 1.17 a	0.68 ± 0.12 a
ONF	4.98 ± 0.03 a	1.27 ± 0.05 a	0.99 ± 0.04 a	5.26 ± 0.49 a	9.34 ± 1.34 a	0.61 ± 0.06 a
PF	4.92 ± 0.09 a	1.32 ± 0.06 a	1.01 ± 0.02 a	5.34 ± 1.04 a	8.43 ± 0.71 a	0.73 ± 0.06 a
PNF	5.03 ± 0.07 a	1.28 ± 0.01 a	1.00 ± 0.03 a	5.19 ± 1.01 a	8.31 ± 1.05 a	0.65 ± 0.05 a
**3 years after land-use conversion (May 2015)**						
OF	4.76 ± 0.05 a	1.26 ± 0.12 a	1.16 ± 0.05 a	7.24 ± 1.44 a	18.82 ± 2.89 a	7.18 ± 1.86 a
ONF	4.87 ± 0.03 b	1.30 ± 0.09 a	1.12 ± 0.06 ab	5.91 ± 1.06 a	13.50 ± 1.21 b	4.33 ± 0.75 b
PF	4.95 ± 0.07 c	1.08 ± 0.06 b	1.08 ± 0.05 b	32.41 ± 6.78 b	13.05 ± 2.21 b	3.53 ± 0.35 b
PNF	5.04 ± 0.04 c	1.05 ± 0.08 b	1.06 ± 0.03 b	11.95 ± 2.15 c	6.67 ± 0.98 c	1.96 ± 0.49 c

### N_2_O fluxes

**Figure 3:**
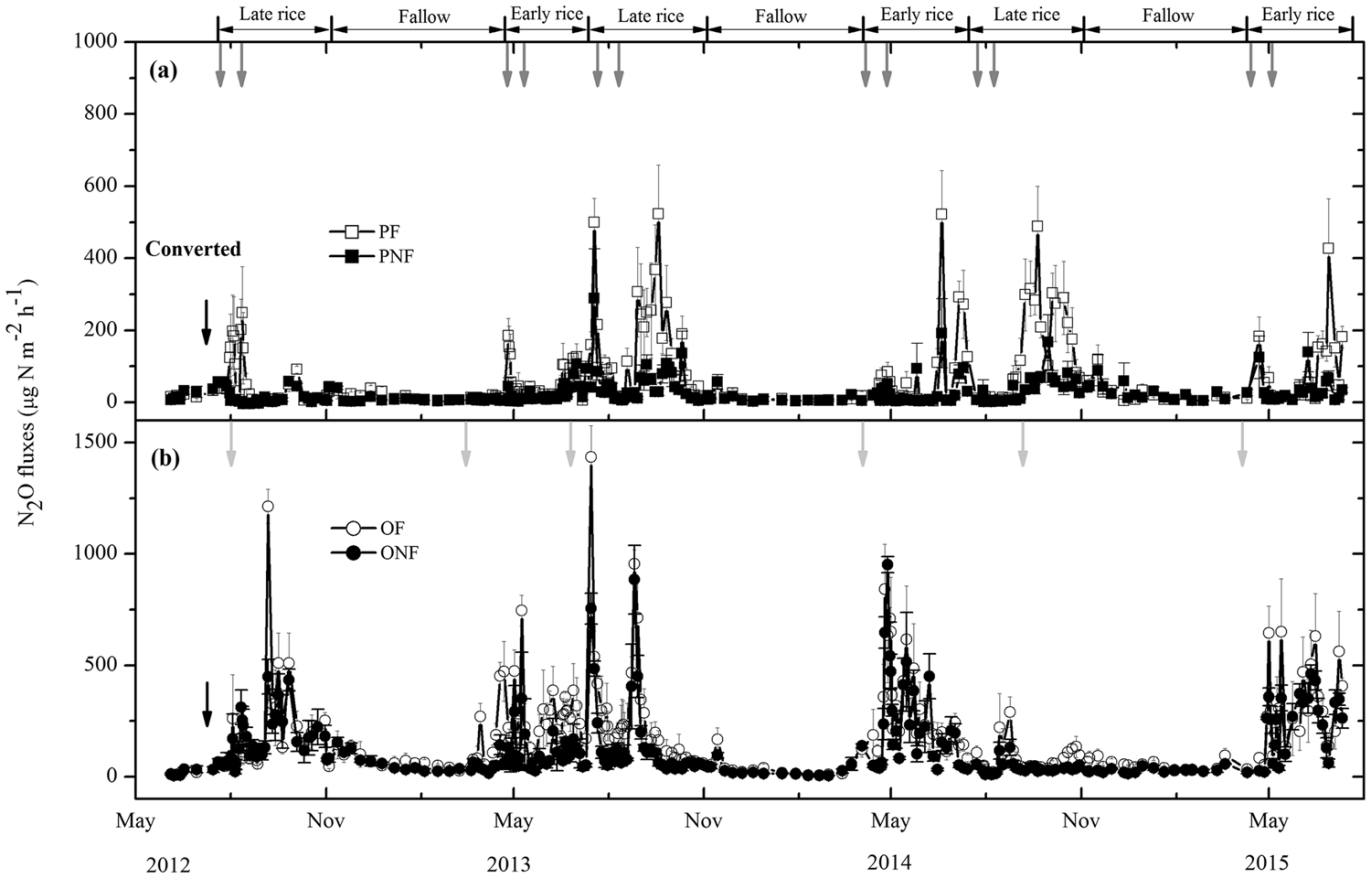
Seasonal dynamics of N_2_O fluxes from conventional paddy fields (**a**) and from a newly converted citrus plantation (**b**) from 2012 to 2015. The data are shown as the means with standard errors. The black arrow indicates the cultivation of orchard and paddy. The grey arrows indicate fertilizer applied to PF, and the light grey arrows indicate fertilizer applied to OF.

**Figure 4:**
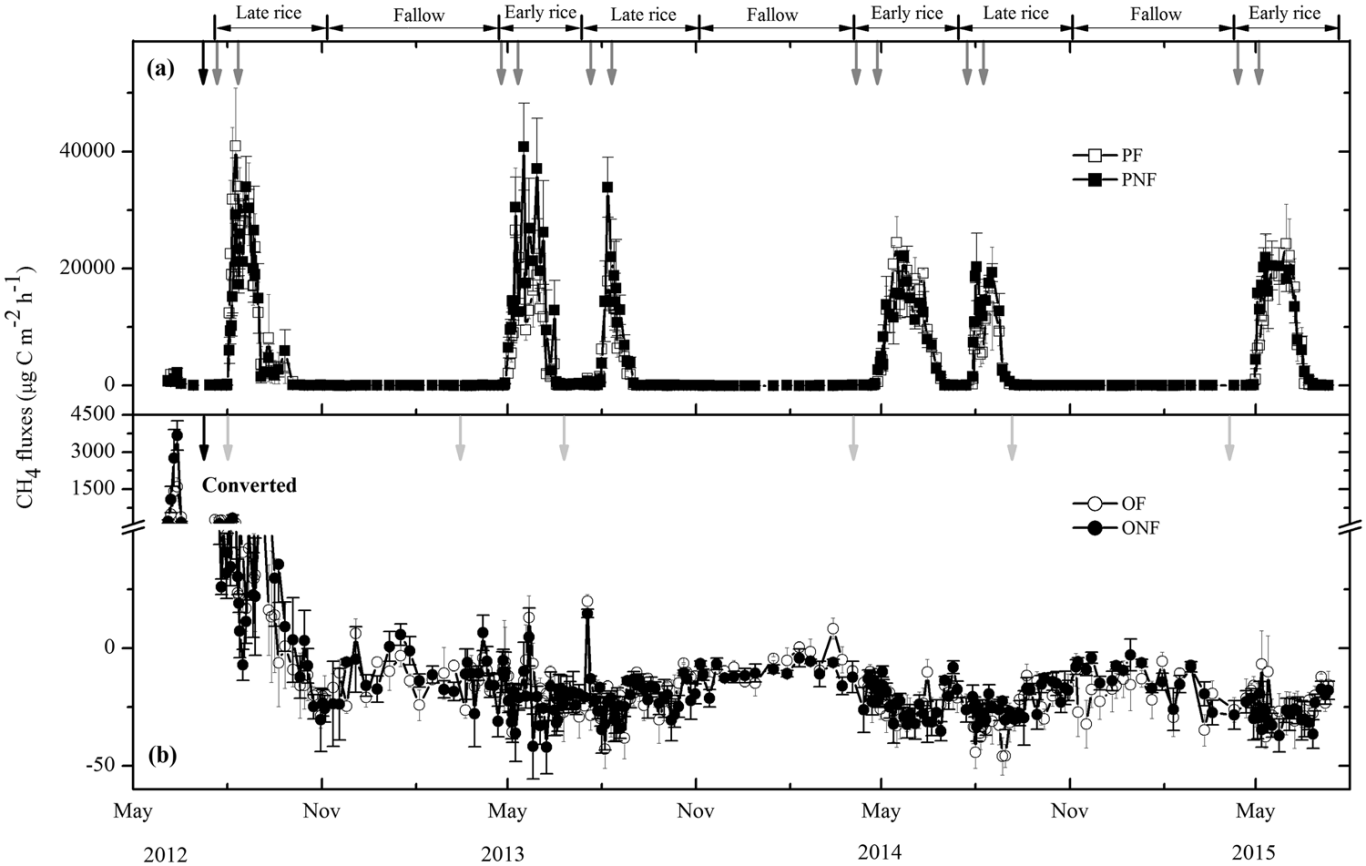
Seasonal dynamics of CH_4_ fluxes from conventional paddy fields (**a**) and newly converted citrus plantation (**b**) from 2012 to 2015. The data are shown as the means with standard errors. The black arrow indicates the cultivation of orchard and paddy. The grey arrows indicate fertilizer applied to PF, and the light grey arrows indicate fertilizer applied to OF.

**Figure 5:**
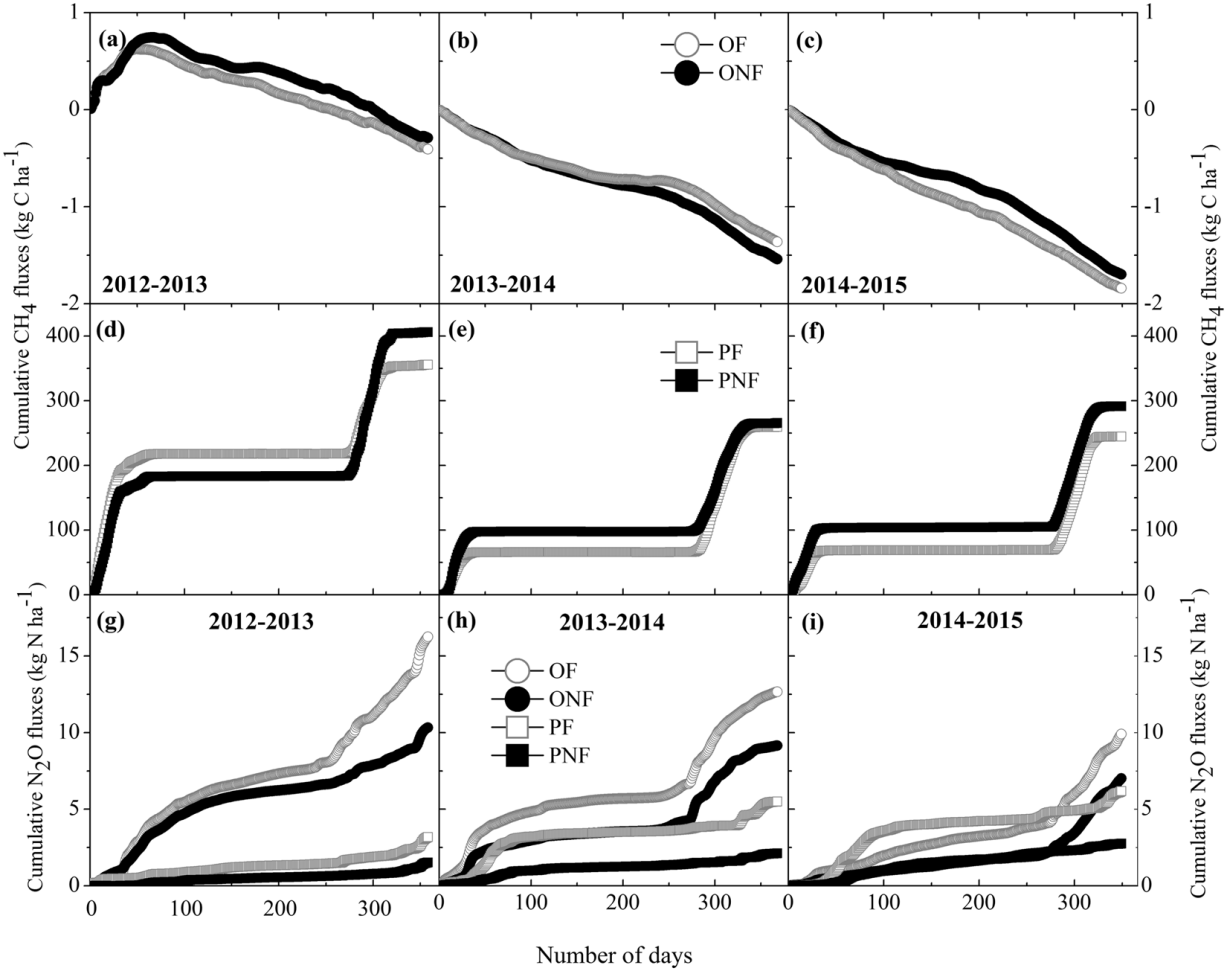
Cumulative CH_4_ (**a**–**f**) and N2O (**g**–**i**) fluxes from all treatments after land-use conversion during each annual cultivation cycle from 2012–2015.

Average N_2_O and CH_4_ fluxes did not differ significantly among the four treatments prior to the land-use conversion (Figs 3 and 4). Seasonal variations in N_2_O flux were characterized by pulse emission events, generally depending on the water irrigation regime and fertilization (Fig. 3). In paddy fields, unperceivable N_2_O fluxes were observed during the flooding periods, whereas substantial emissions occurred after fertilization and at the end of the cropping period, when fields dried off and/or were rewetted by rainfall. Fertilizer application significantly and consistently increased the N_2_O emissions from paddy fields during the three cultivation years (*P* < 0.05), and this stimulating effect of fertilization on N_2_O emissions was enhanced with successive years (Fig. 3a). Over the three cultivation years, the cumulative N_2_O emissions ranged from 3.18 to 6.18 kg N ha^−1^ in the PF, significantly higher than those in the PNF (Fig. 5 and Table 2). As a result, the calculated direct emission factors for N_2_O (EF_d_) from paddy fields were 0.47, 0.94 and 0.96% for the 2012/2013, 2013/2014 and 2014/2015 cultivation years, respectively.

**Table 2:** Cumulative CH_4_ and N_2_O fluxes and CO_2_-eq emissions after land conversion. Numbers in the table represent means with standard errors. OF = orchard with fertilization, ONF = orchard without fertilization, PF = paddy with fertilization, PNF = paddy without fertilization, LC = land-use conversion, and F = fertilization. *P < 0.01, **P < 0.001.

Treatments	CH_4_ flux (kg C ha^−1^)			N_2_O flux (kg N ha^−1^)			CO_2_-eq emission (t CO_2_-eq ha^−1^)		
	2012–2013	2013–2014	2014–2015	2012–2013	2013–2014	2014–2015	2012–2013	2013–2014	2014–2015
OF	−0.41 ± 0.14	−1.36 ± 0.04	−1.84 ± 0.19	16.25 ± 0.66	12.67 ± 1.53	9.91 ± 1.30	7.59 ± 0.31	5.87 ± 0.72	4.56 ± 0.62
ONF	−0.29 ± 0.13	−1.54 ± 0.05	−1.70 ± 0.13	10.33 ± 0.68	9.15 ± 0.61	7.00 ± 0.11	4.82 ± 0.32	4.22 ± 0.29	3.20 ± 0.06
PF	355.83 ± 89.81	259.54 ± 23.53	244.58 ± 51.52	3.18 ± 0.16	5.49 ± 1.65	6.18 ± 1.93	17.62 ± 4.15	14.33 ± 1.84	13.98 ± 3.24
PNF	406.23 ± 57.08	265.72 ± 41.67	291.44 ± 17.60	1.52 ± 0.18	2.12 ± 0.20	2.75 ± 0.16	19.12 ± 2.67	13.04 ± 1.98	14.50 ± 0.87
**Analysis of variance**									
LC	**			**			**		
F	**			**			**		
Year	**			**			**		
LC × F	**			*			*		
LC × Year	**			**			**		
F × Year	**			**			*		
LC × F × Year	**			**			**		

**Figure 6:**
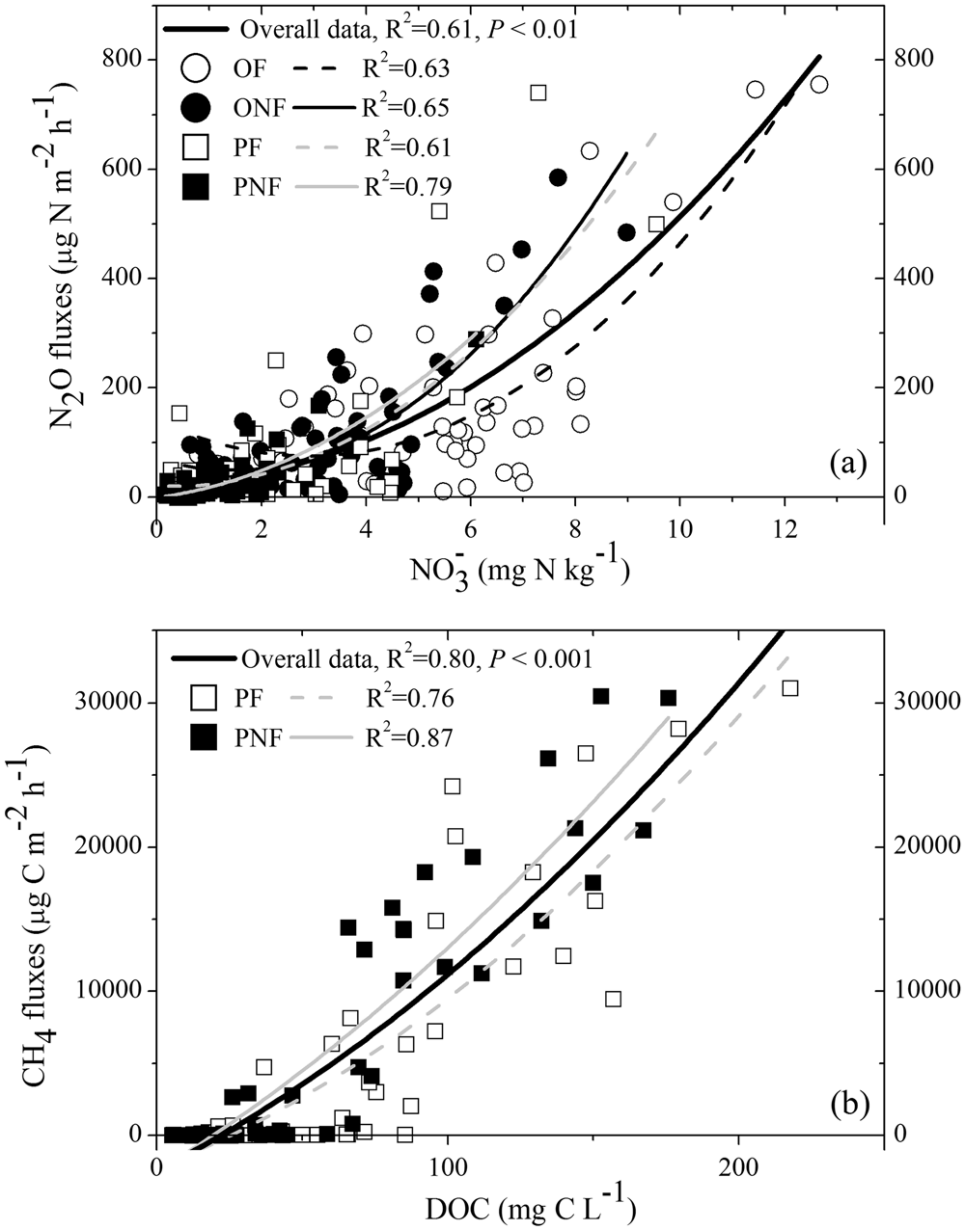
Relationships between N_2_O fluxes from all treatments and soil NO_3_^−^−N content (**a**), and between CH_4_ fluxes from paddy fields and soil DOC concentrations (**b**).

Land-use conversion from rice paddy to citrus orchard significantly increased the N_2_O emissions during the entire observation period (*P* < 0.05, Fig. 3b and Table 2). The high emission peaks in the citrus orchard were mainly linked to fertilization and sharp increases in the soil moisture following irrigation or rainfall events. However, the cumulative N_2_O emissions from the citrus orchard gradually decreased with consecutive cultivation years. Over the entire measurement period, the annual cumulative N_2_O emissions ranged from 7.0 to 10.33 kg N ha^−1^ in the ONF and from 9.91 to 16.25 kg N ha^−1^ in the OF (Fig. 5 and Table 2). Compared to the control, the application of fertilizer in the citrus orchard significantly increased N_2_O emissions (*P* < 0.05, Table 2), and the EF_d_ values were variable for the citrus orchard, ranging between 1.29 and 1.99%. However, in contrast to the rice paddy, the stimulating effect of fertilization on N_2_O emissions in the citrus orchard gradually decreased with successive cultivation years (Table 2). General linear model analysis indicated that the N_2_O emissions were significantly affected by land-use conversion, fertilization and year, as well as by their interactions (*P* < 0.01, Table 2). During the entire observation period, the N_2_O emissions were significantly positively correlated with the soil NO_3_^−^ content for both land-use types (*P* < 0.05, Fig. 6a).

### CH_4_ fluxes

Generally, CH_4_ fluxes from rice paddies were pronounced primarily during the waterlogging stages. Substantial CH_4_ emissions were observed during rice-growing seasons, while no pronounced CH_4_ emissions or a minor sink were observed during the fallow periods (Fig. 4a). During the rice-growing seasons, CH_4_ fluxes increased steadily until the emission peak was attained several weeks after rice transplanting under waterlogging conditions. Thereafter, CH_4_ fluxes decreased dramatically with the drying of fields and decreasing soil water content due to mid-season drainage and then remained at low rates until rice harvest. Although fertilization did not substantially alter the seasonal pattern of CH_4_ fluxes in rice paddies, it significantly decreased the magnitude of CH_4_ emissions (*P* < 0.05, Fig. 4a and Table 2). Over the three cultivation years, the mean annual cumulative CH_4_ emission was 286. 65 kg C ha^−1^ in the PF, which was an average of 12% lower than that in the PNF (Fig. 5 and Table 2). During the entire observation period, the CH_4_ fluxes from rice paddies were significantly positively correlated with the soil DOC concentrations (*P* < 0.05, Fig. 6b).

Land-use conversion from rice paddy to citrus orchard significantly decreased the CH_4_ fluxes during the entire observation period (*P* < 0.05, Fig. 4b and Table 2). There were no regular and consistent seasonal patterns of CH_4_ flux for the citrus orchard during our measurement period. In the newly converted citrus orchard, the soils remained a source of atmospheric CH_4_, which was sustained for several weeks after land-use conversion. Thereafter, CH_4_ emissions gradually decreased, and the soils became a weak sink for atmospheric CH_4_ in the beginning of October 2012, i.e., approximately 2 months after land-use conversion (Fig. 4b). After that, the citrus orchard was generally a sink for CH_4_, with some sporadic CH_4_ emissions during phases with high soil moisture. As a result, cumulative CH_4_ fluxes from the citrus orchard gradually increased with consecutive cultivation years. Over the entire observation period, the annual cumulative CH_4_ fluxes increased from −0.41 to −1.84 kg C ha^−1^ in the OF and from −0.29 to −1.7 kg C ha^−1^ in the ONF (Fig. 5 and Table 2). Compared to the control, there was no significant influence of fertilization on the mean annual CH_4_ fluxes in the citrus orchard. The cumulative CH_4_ fluxes over the entire measurement period varied significantly with land-use conversion, fertilization and year, as well as by their interactions (*P* < 0.01, Table 2).

### Aggregate emissions of CH_4_ and N_2_O

As shown in Table 2, the mean annual CO_2_-eq emissions of CH_4_ and N_2_O were 15.31 and 15.55 t CO_2_-eq ha^−1^ for PF and PNF over the three cultivation years, respectively. These values were 2.5 and 3.8 times higher than those from OF and ONF, respectively, indicating that the land-use conversion of rice paddy field to a citrus orchard significantly decreased the net GHG emissions (*P* < 0.001). The significantly higher CO_2_-eq emissions in rice paddies were mainly due to the substantial CH_4_ emissions during rice-growing seasons. The highest CO_2_-eq emissions in the citrus orchard were observed in the first year after land-use conversion, irrespective of fertilization, after which the annual CO_2_-eq emissions gradually decreased with consecutive cultivation years (Table 2). The application of N fertilizer significantly increased CO_2_-eq emissions in the citrus orchard, mainly due to the substantial increase in N_2_O emissions from the fertilized treatment. However, there was no significant influence of fertilization on the CO_2_-eq emissions in rice paddies, which was largely ascribed to the counteractive effects of stimulated N_2_O emissions and depressed CH_4_ emissions due to fertilization in rice paddies. General linear model analysis indicated that the CO_2_-eq emissions were also significantly affected by land-use conversion, fertilization and year, as well as by their interactions (*P* < 0.01, Table 2).

## Discussion

Over the past decades, numerous measurements of CH_4_ and N_2_O fluxes have been conducted in rice paddies, documenting paddy fields as significant sources of atmospheric CH_4_ and N_2_O^3, 27–29^. However, due to increased demand for livestock products and crop diversification, paddy field-converted upland cultivation systems (e.g., vegetables and orchard) have become increasingly adopted as agricultural systems, especially in the red soil regions of southern China^18, 30^. Although significantly lower CH_4_ but higher N_2_O emissions were generally observed in upland cultivation compared to rice paddies^9, 31^, most previous studies have focused on the comparison of CH_4_ and N_2_O fluxes in different land-use types that have been converted for many years and seldom consider the early stages after land-use conversion^16–19, 31–33^. Recently, several studies have suggested that in addition to accounting for GHG fluxes from specific land-use types, GHG dynamics during actual land-use changes should be also considered^34^, and these studies have recommended that further studies should be designed to monitor the entire conversion process^35, 36^. Although this is an important issue, only a few studies have carried out simultaneous measurements of CH_4_ and N_2_O fluxes during the initial stages after land-use conversion thus far^9, 15^. To our knowledge, the current study is one of the few multi-year *in situ* measurements of CH_4_ and N_2_O fluxes including the initial stages after land-use conversion from rice paddy to upland cultivation in southern China.

The mean annual CH_4_ and N_2_O fluxes from rice paddies in this study were 303.9 kg C ha^−1^ and 3.8 kg N ha^−1^, respectively, over the three cultivation years. These values were within the ranges identified by previous studies in double rice-cropping system with similar fertilization rates^6, 9, 28, 37^, but were much higher than those from other studies in single rice-cropping systems^3, 15, 38^. The mean annual CH_4_ and N_2_O fluxes from the paddy field-converted citrus orchard were −1.19 kg C ha^−1^ and 10.88 kg N ha^−1^, respectively, over the entire study period, which were generally close to previous observations^18, 39, 40^. However, the annual CH_4_ uptake values in the newly converted orchard were significantly lower than those found by Liu *et al*.^20^, who reported an average annual CH_4_ uptake of 2.61 kg C ha^−1^ y^−1^ in a pine plantation in a subtropical region of southern China. This discrepancy might be partly attributed to the difference in the length of time since establishment of the orchard plantation, i.e., newly converted versus 12 years old. Many publications have indicated that soil-atmosphere CH_4_ exchange can be strongly affected by soil disturbances, such as land-use change and agricultural practices, and that these effects may persist for years to decades^41, 42^; this possibility was confirmed by the gradually increasing CH_4_ oxidation capacity after land-use conversion in our study (Fig. 5). In addition, the annual N_2_O emissions from the citrus orchard in this study, especially during the first year after conversion, were generally greater than those from some earlier estimates^20, 39^, probably due to persistent anaerobic conditions during the initial stages after conversion from rice paddy and due to the high amount of basal fertilizer (370 kg N ha^−1^ y^−1^). However, the annual N_2_O emissions from the citrus orchard gradually decreased with consecutive cultivation years due to changes in the soil environmental conditions and management practices. Therefore, our results suggested that long-term, continuous measurements over several years after land-use conversion are needed to provide reliable estimates of the changes in annual CH_4_ and N_2_O fluxes due to land-use change and highlighted the importance of measurements during the initial stages after conversion.

The conversion of rice paddy to citrus orchard significantly reduced CH_4_ emissions and changed the soil from an emission source to sink for atmospheric CH_4_ over the entire measurement period. Although notable quantities of CH_4_ emissions were observed during the initial stages after conversion, the emission rates were significantly lower than those from rice paddies, which were consistent with previous studies showing that CH_4_ emissions can occur during non-flood conditions due to anaerobic microsites^9, 43^. This reduction in CH_4_ emissions after the conversion of rice paddy to citrus orchard can be primarily explained by the shift from anaerobic to aerobic conditions due to improved soil aeration and the regeneration of oxidants, particularly the re-oxidation of Fe(II)^17, 31^. Another driving factor for the reduction in CH_4_ emissions after conversion is the inhibiting effect of aerated conditions on the methanogenic archaeal community. While investigating the abundance of methanogenic archaea at our study site in the years 2013-2014, Liu *et al*. found significantly lower methanogenic archaea abundance in the citrus orchard compared to rice paddies^44^. This finding is in good agreement with results from previous studies that also reported decreasing numbers of methanogens and reductions in resident and active archaea in drained rice paddies^45, 46^. Moreover, significantly higher soil DOC content due to the large quantities of retained crop residues in rice paddies compared to citrus orchards might also contribute to higher CH_4_ emissions in paddy fields because available soil organic C is the predominant source of methanogenic substrates^15, 37^.

In contrast to decreasing CH_4_ emissions, the conversion of rice paddy to citrus orchard resulted in a significant increase in N_2_O emissions in this study. These results are in line with earlier observations showing that the formation of aerobic conditions caused by land-use change can result in reduced CH_4_ fluxes at the expense of increasing N_2_O emissions^9, 47^. Soil moisture is one of the key factors driving N_2_O emissions from many ecosystems due to its role in the stimulation of microbial activity and in the delivery of electron donors and acceptors, as well as in the diffusion of gases in soil^3–5, 14–16^. Although land-use conversion from rice paddy to citrus orchard significantly reduced soil moisture in this study, the strict anaerobic conditions during flooding periods in paddy fields might favour reduction of N_2_O to N_2_ through denitrification processes, thus leading to lower N_2_O emissions^4, 5, 30^. In our study, the N_2_O emissions from rice paddies during the flooding periods were generally low. Substantial emissions only occurred only during periods within several weeks following fertilization and the drying of fields. In addition, the significantly lower soil NO_3_^−^ content and limited nitrate availability under anaerobic conditions might contribute to the lower N_2_O emissions in rice paddies compared to those in citrus orchards, since strong positive correlations between N_2_O emissions and soil NO_3_^−^ content were observed for both land-use types in this study (Fig. 6a). Furthermore, the increased abundance of ammonia-oxidizing archaea in upland cultivations compared to rice paddies could further explain the increasing N_2_O emissions due to enhancement of nitrification processes^46^.

The utilization of synthetic N fertilizers is usually considered an important regulator of CH_4_ and N_2_O fluxes in agriculture fields^5–8, 28^. In this study, the application of N fertilizer generally resulted in a suppression of annual CH_4_ emissions from rice paddies over the 3-year measurement period, which was in accordance with some earlier observations^6, 8, 43^. However, previous studies on the effect of synthetic fertilizers on CH_4_ emissions from rice paddies are inconsistent. Either increased CH_4_ emissions or no significant change in emissions due to fertilization from paddy fields has also been reported in some other studies^28, 30, 48^. In paddy fields, the application of N fertilizer, especially ammonium-based fertilizers, has been found to promote the growth and activity of methane-oxidizing bacteria, especially in soil around rice roots^49^, thus resulting in increased consumption of CH_4_. Moreover, for red soils with sandy loam texture, as in the present study, CH_4_ oxidation under urea-based fertilization is likely further simulated by partially aerobic soil conditions due to the porous and percolating nature of soil^8^. In contrast to rice paddies, no significant effect of fertilization on CH_4_ fluxes was observed in the citrus orchard, which was in agreement with previous studies of upland cultivation areas^30, 50, 51^, probably because both CH_4_ production and oxidation are simultaneously affected by N fertilization^17, 50^. Consistent with numerous previous studies conducted in paddy fields and upland orchard^8, 28, 39^, N_2_O emissions were significantly enhanced by fertilization in both land-use types in this study. These were mainly due to the fact that fertilizer application can markedly increase the soil inorganic content, as also shown in our study (Table 1), thereby providing sufficient substrate for microbial nitrification and denitrification for the production of N_2_O^7–9, 18^. The emission factors for N_2_O were estimated to be 0.47–0.96% and 1.29–1.99% for the rice paddy and citrus orchard, respectively. These results are comparable to previous estimates from paddy fields^5, 8, 25^ and upland orchard^6, 18, 39^. However, large discrepancies in observed EF_d_ for N_2_O in upland orchard, ranging from 0.2–2.2%, have been observed in earlier publications^18, 20, 39, 52^ likely due to the relatively short-term measurements and course sampling intervals, as well as the differences in the usage of the “baseline^39^”, i.e., background emission.

The average annual CO_2_-eq emissions of CH_4_ and N_2_O was 15.43 t CO_2_-eq ha^−1^ for rice paddies over the three cultivation years, similar to values reported in previous studies conducted in the same regions^3, 8, 28, 30^. These values are also within the range of 75–22,237 kg CO_2_-eq ha^−1^ for rice paddies reported by Linquist *et al*.^53^, who estimated aggregate emissions of CH_4_ and N_2_O by collecting 328 measurements globally. However, the annual CO_2_-eq emissions were significantly reduced following the conversion of rice paddies to a citrus orchard (Table 2), indicating that the effect of significantly reduced CH_4_ emissions was only marginally offset by the simultaneously increased N_2_O emissions after land-use conversion. In general, the economic benefits from upland orchard and vegetables were higher than those from rice paddies in our study region^6, 30^. Therefore, lower climate impacts but higher economic incomes can be achieved synchronously by the conversion of rice paddies to citrus orchards in this region. Moreover, the application of fertilizer had no significant effect on the CO_2_-eq emissions in rice paddies, which is consistent with the hypothesis of Zou *et al*.^25^, who surmised that fertilization generally depresses or dose not influence aggregate emissions of CH_4_ and N_2_O from rice paddies, depending on the fertilizer application rate. However, fertilization resulted in significantly higher CO_2_-eq emissions in the citrus orchard, which can be explained by the substantial increase in N_2_O emissions due to higher input of mineral N. It is noteworthy that our analysis of CO_2_-eq emissions did not include the net exchange of CO_2_ between agroecosystems and the atmosphere or changes in soil organic carbon. Results from the literature indicated that rice paddies are usually found to be a weak atmospheric CO_2_ sink^47^ and that carbon sink strength is typically lower than that of mature orchards^54^. Meanwhile, numerous studies have reported that changes in soil organic C are difficult to detect because the magnitude of change is small during several years and because there is a high degree of spatial variation^9, 11^. Therefore, more long-term studies including measurements of the climatically important C- and N-trace gas fluxes (CO_2_, N_2_O and CH_4_) and estimates of changes in soil organic C are needed to provide a complete evaluation of the overall GHG balance during land-use conversion.

In conclusion, this study provided insight into the integrated evaluation of CH_4_ and N_2_O fluxes and their relationships with management practices following the conversion of rice paddies to a citrus orchard over three consecutive cultivation years in a typical red soil region of southern China. Our results not only confirmed that land-use conversion from rice paddy to citrus orchard significantly decreased CH_4_ emissions and increased N_2_O emissions, but also demonstrated that the citrus orchard could persist as a source for atmospheric CH_4_ for several weeks after conversion from paddy fields and then gradually shift to a CH_4_ sink with increasing oxidation capacity over the three cultivation years. Thus, our results highlighted the importance of measurements during the initial stages following land-use conversion and suggested additional long-term continuous observations over several years after conversion. The substantial changes in CH_4_ and N_2_O fluxes following land-use conversion were mainly due to significant alterations in soil environmental conditions (i.e., shifting from anaerobic to aerobic) and soil properties originating from the remarkably different management practices between the two land-use types. Moreover, fertilization significantly increased N_2_O emissions from both land-use types but substantially reduced CH_4_ emissions from the rice paddies and had no significant effect on CH_4_ fluxes in the citrus orchard. As a result, the CO_2_-eq emissions of CH_4_ and N_2_O were significantly reduced following the conversion of rice paddies to citrus orchard, irrespective of fertilization. Overall, reduced aggregate CH_4_ and N_2_O emissions and higher economic benefits can be achieved simultaneously by the conversion of rice paddies to citrus orchards in this red soil region.

## Materials and Methods

### Study site

The experimental site is located at the Qianyanzhou Ecological Research Station (26°44′N, 115°04′E) of the Chinese Academy of Science in Jiangxi Province, southern China. This site has a subtropical monsoon climate with a mean annual air temperature of 18.0 °C and a mean annual precipitation of approximately 1509.0 mm during 1989–2010^6^. The soil type is typical red soil found in middle-subtropical China, classified as Ultisols and some of the Alfisols and Oxisols based on soil taxonomy of the USA^18^. The soil texture is sandy loam, with 58% sand, 31% silt, and 11% clay. Double cropping of rice paddy is the main cropping system in this region, with late rice (late July to late November), a fallow period (late November to next late April), and early rice (late April to late July) in rotation. Other soil properties both before and 3 years after land-use conversion are shown in Table 1.

### Experimental design

The two most prevalent agricultural land uses in our study area were selected, namely, rice paddy (*Oryza sativa L*.) and citrus orchard (*Citrus reticulata*). The experimental site was previously paddy fields for more than 10 years and had been partly converted to a citrus orchard in June 2012. Under each land-use type, two fertilizer treatments (i.e., conventional fertilization and no fertilization) were established. The conventional fertilization treatment followed the local cropping regimes and farmer fertilization practices. The fertilizers used were compound fertilizer (15% N) and urea (46% N). The other treatment was a control without fertilization, with additional management practices being the same as for the fertilization treatment. Therefore, four treatments were established: citrus orchard with fertilization (OF) and without fertilization (ONF) and rice paddy with fertilization (PF) and without fertilization (PNF). All treatments were arranged in a randomized block design with four replicates, for a total of 16 experimental plots (12 × 14 m) that were separated by buffer strips. In the PF, compound fertilizer was applied before rice transplanting, and urea was applied in the form of top dressing at the tillering stage, whereas in the OF, compound fertilizer combined with urea was uniformly spread on the soil surface. To ensure survival and yield, a floodwater layer of 5–7 cm in depth was maintained in the paddy fields until mid-season drainage, and basal fertilizer was amended to a depth of 50 cm before the citrus orchard was established. Details of the cultivation and fertilization practices during the study period are shown in Table S1.

### CH_4_ and N_2_O flux measurement

Fluxes of CH_4_ and N_2_O were simultaneously measured from June 2012 until July 2015 using a static opaque chamber-gas chromatograph (GC) method as described in Yao *et al*.^8^. and Zheng *et al*.^55^. A stainless steel collar (diameter = 40 cm) was pre-installed in the centre of each plot before rice transplanting or orchard planting. The top edge of the collar contains a groove (5 cm in depth) filled with water to seal the rim of a chamber during gas collection. Cylindrical sampling chambers with a diameter of 40 cm and height of 0.39 or 0.69 m (according to the plant height) were covered with a layer of thermal insulation to minimize air temperature changes inside the chamber and equipped with a circulating fan to ensure complete gas mixing during the gas sampling period. The base frames were kept in the same location throughout the entire measurement period in the orchard plots, whereas those in the paddy fields were removed before tillage and placed (24 h before the measurement) in the location marked for subsequent measurements.

Gas samples were taken daily for 5 days after fertilization and once or twice per week for the remaining period and were collected between 09:00–11:00 AM local time. Five air samples were taken from the headspace of each chamber at an interval of 10 min after chamber closure using plastic syringes attached to a three-way stopcock and were stored at room temperature for analysis within a few hours. The chamber headspace temperature was recorded for gas density correction in the flux calculation using a thermometer. The concentrations of CH_4_ and N_2_O were determined by a gas chromatography (Agilent 7890 A, California, USA) equipped with a flame ionization detector (FID) and an electron capture detector (ECD), respectively, as detailed in previous studies^6, 55, 56^. The fluxes were calculated based on the slope of linear or nonlinear regression between concentration and time and were determined as the mean of the four fluxes from the four spatial replications. Annual cumulative CH_4_ and N_2_O fluxes were sequentially computed from the fluxes between every two adjacent intervals of measurements and were estimated by linear interpolation^6, 8^. The fertilizer-induced direct emission factors (EF_d_) for N_2_O were calculated by subtracting the total cumulative emissions of N_2_O in the control treatments from the corresponding cumulative emissions in the fertilized treatments and dividing the result by the fertilizer application rate^5^. For calculating the GHG balance, annual CH_4_ and N_2_O fluxes were converted into CO_2_ equivalents, taking into account the specific radiative forcing potential of 298 for N_2_O and 34 for CH_4_ relative to CO_2_ for a 100-year time horizon^1^.

### Auxiliary measurements

Daily precipitation and air temperature were obtained from the Qianyanzhou meteorological station. The soil temperature and moisture (0–10 cm) for each plot were measured using a potable digital thermometer (JM624, Tianjin, China) and a moisture probe meter (TDR100, Spectrum, USA), respectively. Soil water-filled pore space (WFPS) was calculated from the bulk density (BD) and volumetric soil water content using a particle density of 2.65 g cm^−3^. Floodwater depths in the paddy were measured daily during the flooding period using a ruler. Soil samples (0–10 cm) were collected prior to land-use conversion to determine background information and once per month or every two months between June 2012 and July 2015 for physiochemical property measurements. Soil pH was measured at a soil:water ratio of 1:2.5 using a pH meter. Soil total nitrogen (TN) and dissolved organic carbon (DOC) were determined using an automated C and N analyzer (Elementar, Hanau, Germany). Soil ammonium (NH_4_^+^−N) and nitrate (NO_3_^–^−N) were extracted from 20 g of fresh soil with 1 M KCl (soil:water = 1:5 w/v) and quantified colourimetrically using a flow injection analyzer (Seal AA3, Norderstedt, Germany).

### Statistical analyses

All data are presented as the mean and standard error of mean unless otherwise stated. Analysis of variance (ANOVA) was used to examine differences in soil properties among the four treatments. The impacts of land-use conversion, fertilization and year on CH_4_ and N_2_O fluxes were conducted using general linear models for analysis of variance together with the least significant difference test. The relationships between trace gas fluxes and soil properties were evaluated using a nonlinear regression model. SPSS 20.0 statistical software (IBM Co., New York, USA) was used to conduct statistical analyses. The figures were prepared using Origin 8.5 software (Origin Lab Corporation, USA).

## Electronic supplementary material

Supplementary table
