# Supplementary material for: Responses of CH4 and N2O fluxes to land-use conversion and fertilization in a typical red soil region of southern China
Source: Sci Rep. 2017 Sep 5;7:10571. doi: 10.1038/s41598-017-10806-z (PMC5585344; doi:10.1038/s41598-017-10806-z)
Supplement: Supplementary file 1 — Supplementary table [file 41598_2017_10806_MOESM1_ESM.doc]

**Responses of CH_4_ and N_2_O fluxes to land-use conversion and fertilization in a typical red soil region of southern China**

**Xing Wu****^1,2^, Huifeng Liu^1,3^, Xunhua Zheng^4^, Fei Lu^1^, Shuai Wang^1,2^, Zongshan Li^1,2^, Guohua Liu^1,2^, Bojie Fu^1,2^**

^1^ State Key Laboratory of Urban and Regional Ecology, Research Center for Eco-Environmental Sciences, Chinese Academy of Sciences, Beijing 100085, China.

^2^ Joint Center for Global Change Studies, Beijing 100875, China.

^3^ University of Chinese Academy of Science, Beijing 100049, China.

^4^ State Key Laboratory of Atmospheric Boundary Layer Physics and Atmospheric Chemistry, Institute of Atmospheric Physics, Chinese Academy of Sciences, Beijing 100029, China.

Correspondence and requests for materials should be addressed to X.W. (email: [xingwu@rcees.ac.cn](mailto:xingwu@rcees.ac.cn)) or B.F. (email: bfu@rcees.ac.cn)

**Table S1. Management practices for the experimental fields.**

| Fields | Management | Date |
| --- | --- | --- |
| Orchard | Draining and rotary tillage | Jun 20, 2012 |
|  | Orchard planting, and basal fertilizer (370 kg N ha^-1^) was applied to the fertilized fields | Jul 30, 2012 |
|  | Compound fertilizer (67.2 kg N ha^-1^) and urea (20.8 kg N ha^-1^) was applied to the fertilized fields | Mar 26, 2013 |
|  | Compound fertilizer (67.2 kg N ha^-1^) and urea (20.8 kg N ha^-1^) was applied to the fertilized fields | Jun 17, 2013 |
|  | Compound fertilizer (67.2 kg N ha^-1^) and urea (20.8 kg N ha^-1^) was applied to the fertilized fields | Apr 17, 2014 |
|  | Compound fertilizer (67.2 kg N ha^-1^) and urea (20.8 kg N ha^-1^) was applied to the fertilized fields | Aug 25, 2014 |
|  | Compound fertilizer (67.2 kg N ha^-1^) and urea (20.8 kg N ha^-1^) was applied to the fertilized fields | Apr 4, 2015 |
| Paddy | Rice transplanting, and compound fertilizer (72 kg N ha^-1^) was applied to the fertilized fields | Jul 30, 2012 |
|  | Urea (106.2 kg N ha^-1^) were applied to the fertilized fields | Aug 10, 2012 |
|  | Rice harvest | Nov 14, 2012 |
|  | Rice transplanting, and compound fertilizer (72 kg N ha^-1^) was applied to the fertilized fields | Apr 24, 2013 |
|  | Urea (106.2 kg N ha^-1^) were applied to the fertilized fields | May 3, 2013 |
|  | Rice harvest | Jul 22, 2013 |
|  | Rice transplanting, and compound fertilizer (72 kg N ha^-1^) was applied to the fertilized fields | Jul 30, 2013 |
|  | Urea (106.2 kg N ha^-1^) were applied to the fertilized fields | Aug 10, 2013 |
|  | Rice harvest | Nov 14, 2013 |
|  | Rice transplanting, and compound fertilizer (72 kg N ha^-1^) was applied to the fertilized fields | Apr 19, 2014 |
|  | Urea (106.2 kg N ha^-1^) were applied to the fertilized fields | Apr 29, 2014 |
|  | Rice harvest | Jul 20, 2014 |
|  | Rice transplanting, and compound fertilizer (72 kg N ha^-1^) was applied to the fertilized fields | Jul 28, 2014 |
|  | Urea (106.2 kg N ha^-1^) were applied to the fertilized fields | Aug 8, 2014 |
|  | Rice harvest | Nov 11, 2014 |
|  | Rice transplanting, and compound fertilizer (72 kg N ha^-1^) was applied to the fertilized fields | Apr 26, 2015 |
|  | Urea (106.2 kg N ha^-1^) were applied to the fertilized fields | May 6, 2015 |
|  | Rice harvest | Jul 20, 2015 |
